# TECHNOSOCIAL INNOVATIONS IN THE HEALTHCARE SECTOR: WHAT ETHICAL ISSUES MIGHT WE FACE AS WE AGE?

**DOI:** 10.1093/geroni/igad104.3621

**Published:** 2023-12-21

**Authors:** Marie-Michele Lord, Marie-Josee Drolet

**Affiliations:** Université du Québec à Trois-Rivières, Trois-Rivieres, Quebec, Canada; Université du Québec à Trois-Rivières, Trois-Rivieres, Quebec, Canada

## Abstract

Technosocial innovations are designed to transcend current practices by linking technological development to community well-being. Their dazzling development is gradually transforming health services. Despite their recognized potential, a growing number of studies point to the ethical risks associated with their development. We are currently conducting a study to obtain a comprehensive picture of the ethical issues associated with the use of technosocial innovations for older adults and their care partners in the healthcare sector, and to identify key indicators of the presence of those ethical issues. A prospective design is use and combines a systematic literature review with a Delphi method. Of all the studies looking at the ethical issues involved in deploying technosocial innovations in the healthcare sector, a limited number have focused on those we may experience as we age. Still, six empirically-supported ethical issues have emerged : 1) exacerbation of the digital divide; 2) exclusion at multiple levels; 3) epistemic injustices; 4) infringement of dignity and confidentiality; 5) ageism; and 6) increased dependency dynamics. A number of indicators were identified in the early stages of the Delphi method, including a high rate of abandonment of the use of proposed innovations, and performance indicators with little integration of PREMs and PROMs. It is imperative to translate current knowledge into concrete resources that can be used in order to protect our rights and health as we age. This study is a first step towards identifying indicators that can be concretely used by health organizations wishing to act ethically.

